# Myeloid-Specific *Rictor* Deletion Induces M1 Macrophage Polarization and Potentiates *In Vivo* Pro-Inflammatory Response to Lipopolysaccharide

**DOI:** 10.1371/journal.pone.0095432

**Published:** 2014-04-16

**Authors:** William T. Festuccia, Philippe Pouliot, Inan Bakan, David M. Sabatini, Mathieu Laplante

**Affiliations:** 1 Department of Physiology and Biophysics, Institute of Biomedical Sciences, University of São Paulo, São Paulo, Brazil; 2 Whitehead Institute for Biomedical Research, Massachusetts Institute of Technology, Cambridge, Massachusetts, United States of America; 3 Howard Hughes Medical Institute, Massachusetts Institute of Technology, Massachusetts, United States of America; 4 Koch Center for Integrative Cancer Research at MIT, Massachusetts Institute of Technology, Cambridge, Massachusetts, United States of America; 5 Centre de Recherche de l’Institut Universitaire de Cardiologie et de Pneumologie de Québec (CRIUCPQ), Université Laval, Québec, Québec, Canada; Virginia Polytechnic Institute and State University, United States of America

## Abstract

The phosphoinositide-3-kinase (PI3K)/protein kinase B (Akt) axis plays a central role in attenuating inflammation upon macrophage stimulation with toll-like receptor (TLR) ligands. The mechanistic target of rapamycin complex 2 (mTORC2) relays signal from PI3K to Akt but its role in modulating inflammation *in vivo* has never been investigated. To evaluate the role of mTORC2 in the regulation of inflammation *in vivo*, we have generated a mouse model lacking *Rictor*, an essential mTORC2 component, in myeloid cells. Primary macrophages isolated from myeloid-specific *Rictor* null mice exhibited an exaggerated response to TLRs ligands, and expressed high levels of M1 genes and lower levels of M2 markers. To determine whether the loss of *Rictor* similarly affected inflammation *in vivo*, mice were either fed a high fat diet, a situation promoting chronic but low-grade inflammation, or were injected with lipopolysaccharide (LPS), which mimics an acute, severe septic inflammatory condition. Although high fat feeding contributed to promote obesity, inflammation, macrophage infiltration in adipose tissue and systemic insulin resistance, we did not observe a significant impact of *Rictor* loss on these parameters. However, mice lacking *Rictor* exhibited a higher sensitivity to sceptic shock when injected with LPS. Altogether, these results indicate that mTORC2 is a key negative regulator of macrophages TLR signalling and that its role in modulating inflammation is particularly important in the context of severe inflammatory challenges. These observations suggest that approaches aimed at modulating mTORC2 activity may represent a possible therapeutic approach for diseases linked to excessive inflammation.

## Introduction

The innate immune system plays a fundamental role in protecting organisms against a variety of infectious agents. Macrophages are a heterogeneous group of cells with phagocytic activity that are fulfilling key roles in innate immunity[1]. Macrophages digest and kill pathogens and helps maintain tissue homeostasis by removing dead cells and debris. In response to pathogens, these cells produce several pro-inflammatory cytokines and chemokines that contribute to infection clearance. Over the last decades, various macrophage subsets with distinct immune functions have been described[2]. Classically activated macrophages (M1 macrophages) have pro-inflammatory functions and mediate host defense against various pathogens and exert anti-tumor immune responses. Alternatively activated macrophages (M2 macrophages), on the other hand, exhibit anti-inflammatory roles and regulate tissue homeostasis in processes like wound healing.

Toll-like receptors (TLR) are molecular pattern recognition receptors recognizing danger signals derived from a plethora of infectious agents [3]. Macrophages express these receptors and react to such receptor ligation. Indeed, upon ligand binding, TLR molecules recruit signaling adapters and initiate a pro-inflammatory response that culminates with the activation of several transcription factors that turn on the expression of pro-inflammatory genes required for pathogen clearance. Although the importance of TLR signalling in immune defense is well known, an exaggerated inflammatory response can also seriously impair the functions of tissues and lead to sceptic shock and death. Uncontrolled activation of TLR in macrophages contributes to the development of autoimmune diseases and atherosclerosis and promotes the susceptibility to tumor metastasis[4]. Along with cancer, chronic low-grade inflammation plays a critical role in the development of obesity and insulin resistance[5]. In this context, gaining insights into the regulation of inflammation could provide new therapeutic approaches to treat human pathologies.

Class I phosphoinositide-3-kinase (PI3K) is a class of lipid kinases that plays diverse roles in cellular functions such as cell growth, proliferation, differentiation, motility, survival and intracellular trafficking[6], [7]. Upon activation, PI3K is recruited to the plasma membrane, where it phosphorylates phosphatidylinositol (4,5)-biphosphate (PIP2) to generate phosphatidylinositol (3,4,5)-triphosphate (PIP3). Increased PIP3 levels promotes the recruitment of Akt (also known as protein kinase B) to the plasma membrane, causing Akt phosphorylation by 3-phosphoinositide-dependent protein kinase 1 (PDPK1) on threonine 308 (Thr308) and by the mechanistic target of rapamaycin (mTOR) complex 2 (mTORC2) on serine 473 (Ser473)[8]. These phosphorylation events, that are required for a maximal activation of Akt, allow this kinase to regulate processes including metabolism and survival[9]. Several reports show that the PI3K-Akt axis is a key regulator of the cellular response to TLR ligands[10]–[12]. Activated TLRs physically recruit and activate PI3K[13]–[17], promoting an elevation in Akt phosphorylation[13], [17]–[21]. Many studies indicate that PI3K-Akt negatively regulates the expression of several pro-inflammatory genes in response to TLR ligands in macrophages *in vitro* [10]–[12]. Consistent with these results, macrophages isolated from myeloid-specific *Pdpk1* knockout mice show a reduction in Akt phosphorylation and an exaggerated pro-inflammatory response to LPS[22]. The fact that *Akt1* deficient macrophages display elevated cytokine production and that mice lacking *Akt1* have impaired tolerance to LPS stimulation support the idea that this kinase is a critical element regulating TLR signalling downstream of PI3K[23], [24].

The mechanistic target of rapamycin is a key component of a signaling network that senses and integrates a variety of environmental cues to regulate organismal growth and homeostasis[25], [26]. This serine/threonine protein kinase interacts with several proteins to form two distinct complexes named mTOR complex 1 (mTORC1) and mTORC2. mTORC2 is activated by growth factors such as insulin through yet poorly defined mechanism(s) requiring PI3K. When active, mTORC2 phosphorylates Akt, serum- and glucocorticoid-induced protein kinase 1 (SGK1), and protein kinase C-α (PKC-α). The role of mTORC2 in modulating TLR signalling in macrophages is still unclear. Since mTORC2 deletion is shown to impair Akt action towards some, but not all of its substrates[27], it is difficult to predict its implication in the regulation of inflammation. An elegant study published recently has demonstrated that the reduction in mTORC2 function exacerbates the pro-inflammatory response to LPS in dendritic cells (DC) *in vitro* [18]. The same report revealed that mTORC2 modulates inflammation through Akt activation and Forkhead box O1 (FoxO1) nuclear localization. Whether mTORC2 has a similar impact in macrophages and, most importantly, how its function affects the inflammatory response to acute and chronic inflammation in a physiological context remains to be characterized.

In this report, we show in primary macrophages and cell lines *in vitro* that the activation of TLRs promotes mTORC2 activity. In order to determine the role of mTORC2 in the macrophage regulation of the inflammation *in vivo*, we have generated a mouse model lacking the mTORC2 component rapamycin-insensitive companion of mTOR *(Rictor)* in myeloid cells. Macrophages isolated from these mice exhibit an exaggerated response to TLRs ligands, and express high levels of M1 genes and lower levels of M2 markers. Surprisingly, loss of *Rictor* did not exacerbate macrophage infiltration in adipose tissue, inflammation and insulin resistance when animals were exposed to a high fat diet, a condition known to promote a chronic, but low-grade inflammatory state. However, mice lacking *Rictor* exhibited a higher sensitivity to sceptic shock when injected with LPS. Together, these results indicate that mTORC2 is a key regulator of TLR signalling that plays key roles in the down-regulation of the inflammatory response during acute infection.

## Materials and Methods

### Cell Lines, Antibodies, and Reagents

Reagents were obtained from the following sources: antibodies to phospho-S473 Akt, phospho-T308-Akt, Akt, mTOR, RICTOR, NDRG1, phospho-T346-NDRG1, phospho-PKCαβ-T636/641, phospho-Thr180/Tyr182-p38, phospho-S180/S181-IKKα/β, phospho-Thr202/Tyr204-Erk1/2, phospho-Thr183/Tyr185-JNK from Cell Signaling Technology (cat #4060, 2965, 4691, 2983, 2140, 5196, 3317, 9375, 9211, 2681 and 9101 respectively); antibodies to PKCα and IκBα from Santa Cruz Biotechnology (cat #SC-208 and SC-371). Secondary antibodies were all purchased from Santa Cruz Biotechnology. TLR ligands were purchased from Sigma (LPS, L3012), Integrated DNA technology (CpG), Enzo Life Sciences (Malp2, ALX-162-027-C5050; PAM3, ALX-165-066-M022; and R848, ALX-420-038-M005). Primary mouse fibroblasts were established from E13.5 embryos of *Rictor* ^Lox/Lox^ mice crossed with P53^−/−^ mice, as described previously[27]. Raw264.7 macrophages were purchased from ATCC.

### Isolation and Culture of TEM

This study was carried out in accordance with the recommendations in the Guide for the Care and Use of Laboratory Animals of the National Institutes of Health. The protocol was approved by the Massachusetts Institute of Technology Committee on Animal Care (CAC) (permit 090908012). For thioglycollate-elicited macrophages (TEM) isolation, mice were injected intraperitoneally (ip) with 1.0 mL of a sterile solution of thioglycollate (3%) (BD Bioscience). Four days later, mice were sacrificed and macrophages collected by a peritoneal cavity wash under aseptic conditions with 10 mL of sterile PBS. Next, macrophages were plated in 5% CO^2^ humidified incubator at 37°C for 1 hour in RPMI 1640 media supplemented with 10% heat inactivated fetal calf serum (FCS), 1% penicillin-streptomycin (Gibco, 15140-122) and 2 mM L-glutamine (Gibco, 35050-061). Plates were then washed 5 times with warm PBS to remove non-adherent cells. TEM were allowed to sit for 24 hours and then used for experimentation.

### Isolation, Differentiation, and Culture of BMDM

For bone marrow derived-macrophage (BMDM) isolation, mice were sacrificed and both tibias and femur were collected. Skin and muscles were removed and the bones were washed in 70% ethanol. Bone marrow was flushed out the bones using a syringe filled with BMDM growth media. The base of this medium is the same that the one used for TEM, but supplemented with 30% of L-Cell conditioned media. To make L-Cell conditioned media, 2.5×10^5^ L929 fibroblasts (ATCC) were plated into 50 ml of RPMI 1640 media containing 10% FCS, penicillin-streptomycin and glutamine as described above in T175 flasks. Cells were grown to confluence (5 days) and media was centrifuged, filtered and store frozen in aliquots at −80 C until needed. The bone marrow cells isolated from the tibias and femurs were resuspended in BMDM growth media and the cells allowed to differentiate for 6 days. Subsequently, media was changed every two days.

### Generation of Macrophage-specific Rictor Null Mice

*Rictor* ^Lox/Lox^ mice were produced as described before[28]. These animals were backcrossed 4 times to C57BL/6J and were then crossed to *LysM-cre* ^+/+^ transgenic mice [B6.129P2-*Lyz2tm1(cre)Ifo*/J, Jackson Laboratory] to obtain heterozygous *LysM-cre* ^+/−^; *Rictor* ^Lox/Wt^ offspring (where WT refers to wild type) as F1 generation[29]. These heterozygous mice were crossed to obtain the LysM-*Rictor* ^KO^ with genotype *LysM-cre* ^+/?^; *Rictor* ^Lox/Lox^ and their wild-type littermates that are *LysM-cre* ^−/−^ (referred to here as *LysM-Rictor* ^WT^). For all mice used, the genotypes were determined by PCR analysis of tail genomic DNA as described previously[29].

### Metabolic Studies with *LysM-Rictor^WT^* or *LysM-Rictor^KO^* Mice

Mice (6–8 weeks old) were fed a low fat diet (10% kcal fat, Research Diet, D12450B) or high fat diet (60% kcal fat, Research Diet, D12492) for 21 and 23 weeks respectively. The mice were maintained under temperature- and humidity-controlled conditions with a 12-h light/dark cycle and were allowed to eat and drink water ad libitum. At week 18, mice were fasted overnight before a glucose tolerance test (GTT) on the following morning. Briefly, blood was collected from the tail at time 0, 15, 30, 45, 60 and 120 minutes following a ip injection of glucose (1g/kg). Glucose levels were measured using a Lifescan OneTouch Ultra glucose meter. At week 19, mice were fasted for 6 hours before the insulin tolerance test. Blood was collected from mouse tail 0, 15, 30, 45, 60 and 120 minutes following an ip injection of insulin (0.75U/kg, Eli Lilly) and glucose levels were measured as described for the GTT. Mice were then allowed to rest for few 2 to 4 weeks. On the day of sacrifice, following an overnight fast, the animals were anaesthetized with isoflurane and killed by cardiac punction in accordance with the ethical guidelines. Tissues were collected and frozen for analysis. Plasma metabolites were measured using commercial kits from different companies; insulin (Ultra sensitive Mouse Insulin ELISA kit, Crystal Chem Inc.), triglycerides (Infinity Triglycerides, Thermo Scientific), and cholesterol (Infinity Total Cholesterol, Thermo Scientific).

### *In vivo* Injection of LPS

*LysM*-*Rictor* ^WT^ or *LysM-Rictor* ^KO^ mice (6–8 months old) were injected ip with a solution of LPS (2.5 mg/kg) (Sigma Aldrich L3012). Body temperature was monitored over the course of the experimentation using a rectal temperature sensor. Blood was collected before and after LPS injection. Preliminary experiments carried in our laboratory showed that mice having a core body temperature dropping down to 33+/−0.2°C or below, following LPS injection, have a poor recovery rate and a high probability of death and this was set as a criteria for an ethical sacrifice: mice reaching this limit were humanely sacrificed by CO^2^ asphyxiation.

### Protein Lysates Preparation for Western Blotting

After washing with ice-cold PBS, cells were lysed with Triton-X 100 containing lysis buffer (50 mM HEPES [pH 7.4], 2 mM EDTA, 10 mM sodium pyrophosphate, 10 mM sodium glycerophosphate, 40 mM NaCl, 50 mM NaF, 2 Mm sodium orthovanadate, 1% Triton-X 100, and one tablet of EDTA-free protease inhibitors (Roche) per 25 ml). Cells were then rotated at 4°C for 10 minutes before the soluble fraction of cell lysates were isolated by centrifugation (13,000 rpm, 10 min). Protein levels were quantified using Bradford reagents (BioRad) and analyzed by western blotting using ECL (GE healthcare Life Sciences).

### RNA Isolation and qPCR Analysis

Total RNA was isolated from cells or tissues using the RNeasy Kit (Qiagen) and reverse-transcription was performed using Superscript III reverse transcriptase (Invitrogen). The resulting cDNA was diluted in DNase-free water (1∶15) before quantification by real-time PCR. mRNA transcript levels were measured using SYBR Green PCR master mix (Applied Biosystems) and the Biosystems 7900HT Sequence Detection System v2.3 software. All Data are expressed as the ratio between the expression of target gene to the housekeeping genes 36B4.

### Cytokine Measurements

Cytokines were measured from cell culture media and plasma using commercial ELISA kits from R&D according to manufacturer’s instructions.

### Statistical Analysis

Results are expressed as means ± SE. When appropriated, Student’s unpaired t tests or factorial ANOVA followed by Newman-Keuls’ multiple range tests were used for multiple comparisons. *P*<0.05 was taken as the threshold of significance.

## Results

**Figure 1:**
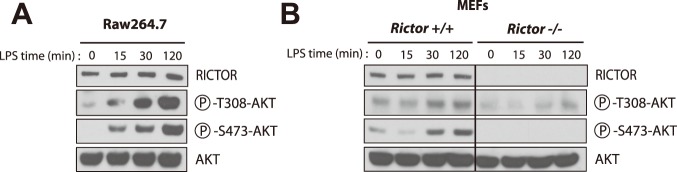
TLR4 activation with LPS induces the phosphorylation of AKT. (A) Response of Raw264.7 macrophages to LPS. Raw264.7 macrophages were plated and incubated overnight. Cells were then washed twice with PBS and incubated for 16 hours in DMEM 0.1% FBS. Then, cells were treated with LPS (500ng/ml) for the indicated time. Proteins were extracted and lysates were analysed by immunoblotting for indicated proteins. (B) Impact of LPS on cell signalling in *Rictor* ^+/+^ and *Rictor* ^−/−^ MEFs. MEFs were plated 24 hours before the experimentation. The next day, cells were washed twice with PBS and incubated in DMEM 0.1% FBS for 2 hours. Cells were then treated with LPS (5ug/ml) for the indicate time. Proteins were extracted and lysates were analysed by immunoblotting for indicated proteins.

Over the years, several groups observed that TLR stimulation increases the activation of the PI3K-Akt axis[13], [17]–[21]. Supporting these findings, we observed that stimulation of Raw264.7 macrophages with the TLR4 agonist LPS rapidly enhances Akt phosphorylation (Figure 1A). Interestingly, LPS not only induced the phosphorylation of Akt on Thr308, a site phosphorylated by PDPK1[30], but also induced the phosphorylation of Ser473, a site phosphorylated by mTORC2[31], suggesting the implication of mTORC2 in the cellular response to TLR stimulation. In order to characterize the implication of mTORC2 in the regulation of PI3K-Akt following TLR activation, we stimulated *Rictor^−/−^* mouse embryonic fibroblasts (MEFs) with LPS. Similarly to what was observed in Raw264.7 macrophages, LPS induced the phosphorylation of Akt on Thr308 and Ser473 residues in wild-type MEFs (Figure 1B). Interestingly, loss of *Rictor* completely abrogated the phosphorylation of Ser473, while the phosphorylation of Akt on Thr308 was only partially reduced. These results indicate that mTORC2 plays a necessary role in TLR4-mediated activation of Akt.

**Figure 2:**
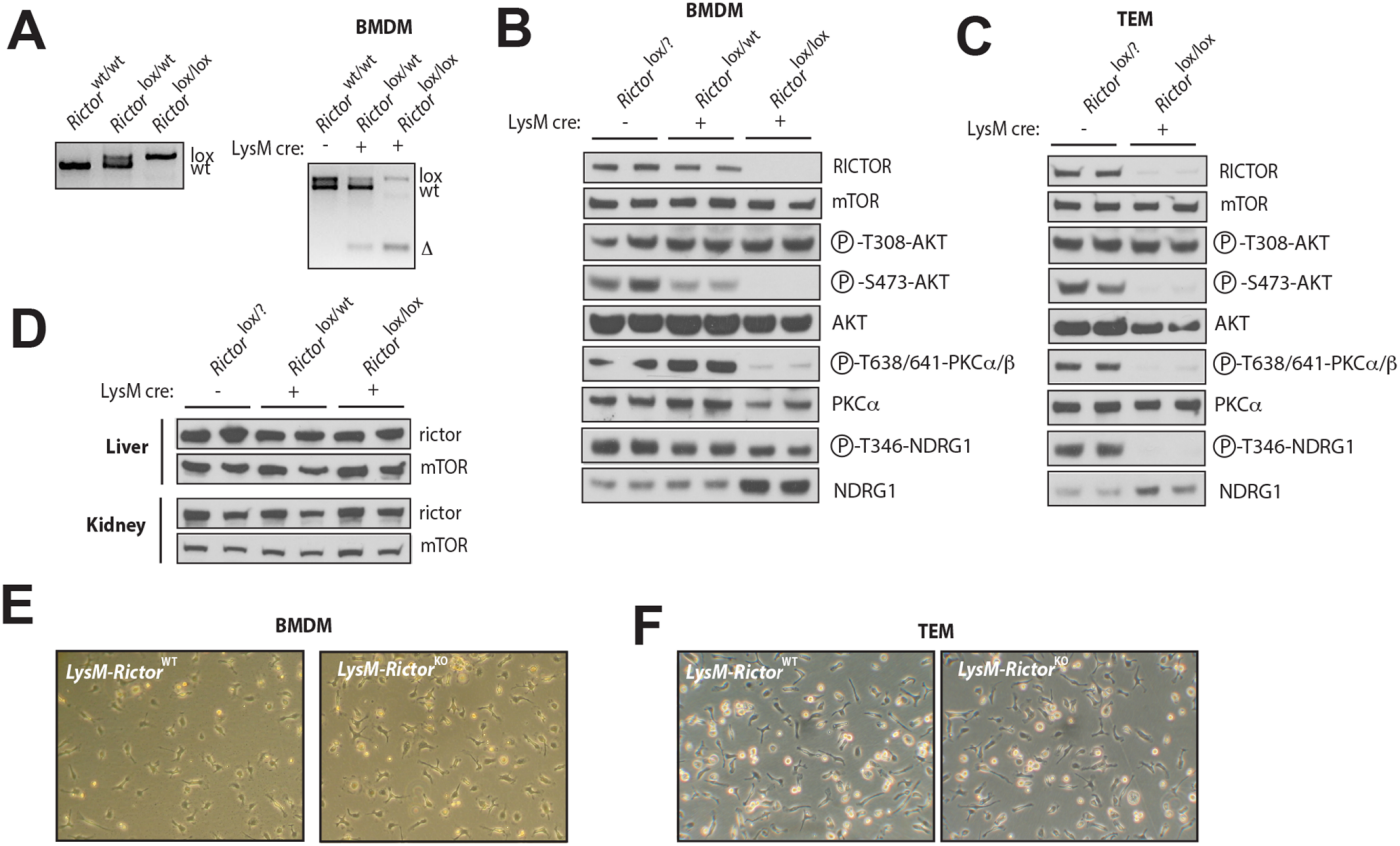
Production of a macrophage-specific *Rictor* knock-out mouse model. (A) PCR strategy for the genotyping and identification of wild-type and KO mice. On the left panel is presented the PCR reaction showing the presence or the absence of the Lox allele in the *Rictor* gene. PCR was performed from a piece of tail collected from mice. The right part of the panel shows the efficiency of the recombination of the *Rictor* allele in BMDM isolated from the *Rictor* ^wt/wt^, *Rictor* ^Lox/wt^, *Rictor* ^Lox/Lox^ expressing or not the LysM^cre^. The presence of the Δ allele confirms the recombination of the Lox sites and the deletion of the targeted exon. (B to D) Confirmation of the specific loss of RICTOR in (B) BMDM and (C) TEM but not in (D) other mouse tissues. BMDM and TEM were isolated from mice and cultured as described in the methods sections. Proteins were extracted from cells and tissues and lysates were analysed by immunoblotting for indicated proteins. In B, C, and D, each line represents one mouse. (E–F) The loss of *Rictor* does not affect cell morphology and proliferation of (E) BMDM or (F) TEM.

Observing that mTORC2 regulates elements of the TLR signalling, we next evaluated the implication of mTORC2 in regulating the functionality of macrophages *in vitro* and *in vivo*. To do so, we crossed *Rictor* ^Lox/Lox^ mice[28] with mice expressing a Cre-recombinase under the control of the Lysozyme M promoter (*LysM* ^Cre^ mice)[29]. This strategy allowed the selective depletion of *Rictor* in myeloid cells (granulocytes and mature macrophages) (*LysM*-*Rictor* ^KO^)[29]. *LysM*-*Rictor* ^KO^ mice were born at the expected Mendelian ratio and did not show any obvious abnormality. As shown in Figure 2, the presence of *LysM* ^Cre^ in *Rictor* ^Lox/Lox^ mice resulted in the recombination of *Rictor* gene and in the loss of the protein in bone marrow-derived macrophages (BMDM) and in thyoglycollate-elicited macrophages (TEM) (Figure 2A, 2B and 2C). Confirming the specificity of our approach, we did not observe any change in RICTOR protein levels in the peripheral tissues of *LysM*-*Rictor* ^KO^ mice (Figure 2D). As expected, *Rictor* loss severely impaired the phosphorylation of Akt on Ser473 in a dose-dependent manner (Figure 2B and 2C). We also observed that the phosphorylation of protein kinase Cα/β (PKCα/β) was significantly reduced in the BMDM and the TEM of *LysM*-*Rictor* ^KO^ mice (Figure 2B and 2C). The phosphorylation of N-myc downstream-regulated gene 1 (NDRG1), a marker of the activation state of the mTORC2 substrate SGK1[32], was also greatly reduced in TEM isolated from *LysM*-*Rictor* ^KO^ mice (Figure 2C). Surprisingly, NDRG1 phosphorylation was only partially reduced in BMDM of *LysM*-*Rictor* ^KO^ mice (Figure 2B). This effect is likely a consequence of a compensatory mechanism, as we observed that total NDRG1 levels rose significantly is response to *Rictor* loss. Lastly, although mTORC2 was shown to regulate cell survival, metabolism, and cytoskeleton organization through its impact on various AGC kinases[25], we did not observed any impact of *Rictor* depletion on *in vitro* macrophage differentiation and morphology (Figure 2E and 2F).

**Figure 3:**
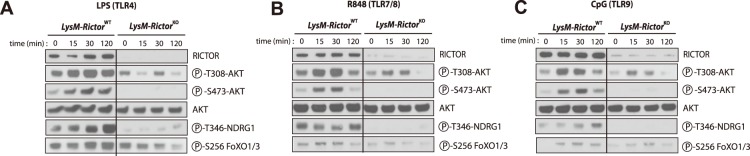
TLR activation promotes AKT phosphorylation through the activation of mTORC2. BMDM were isolated from LysM-*Rictor* ^WT^ or LysM-*Rictor* ^KO^ mice and were differentiated for 6 days *in vitro*. Cells were then plated and incubated 24 hours. Cells were wash twice with PBS, incubated 2 hours in RPMI 0.1% FBS and then treated with (A) LPS (250ng/ml), (B) R848 (0.1uM), or (C) CpG (0.5uM) for the indicated times. Proteins were extracted and lysates were analysed by immunoblotting for indicated proteins.

In order to investigate a possible involvement of mTORC2 in the pro-inflammatory response of macrophages to TLR stimulation, we treated BMDM isolated from either *LysM*-*Rictor* ^WT^ or *LysM*-*Rictor* ^KO^ mice with TLR4 (LPS), TLR7/8 (R848), or TLR9 (CpG) ligands. As observed in Raw 264.7 and MEFs (Figure 1), activation of TLR in BMDM led to a significant rise in Akt phosphorylation (both Thr308 and Ser473)(Figure 3A, 3B, and 3C). Interestingly, *Rictor* loss abolished Akt phosphorylation on Ser473 and partially blocked its phosphorylation on Thr308, emphasizing the need of mTORC2 for the maximal activation of Akt. We also noted that the phosphorylation of NDRG1 and FoxO1/3 was increased in response to TLR, an effect that was also reduced in *Rictor* null cells. These results clearly show that mTORC2 is activated in response to TLR stimulation in macrophages. Lastly, as shown many times by others[18], [27], [33], we observed no effect of *Rictor* loss on the phosphorylation of GSK3β.

**Figure 4:**
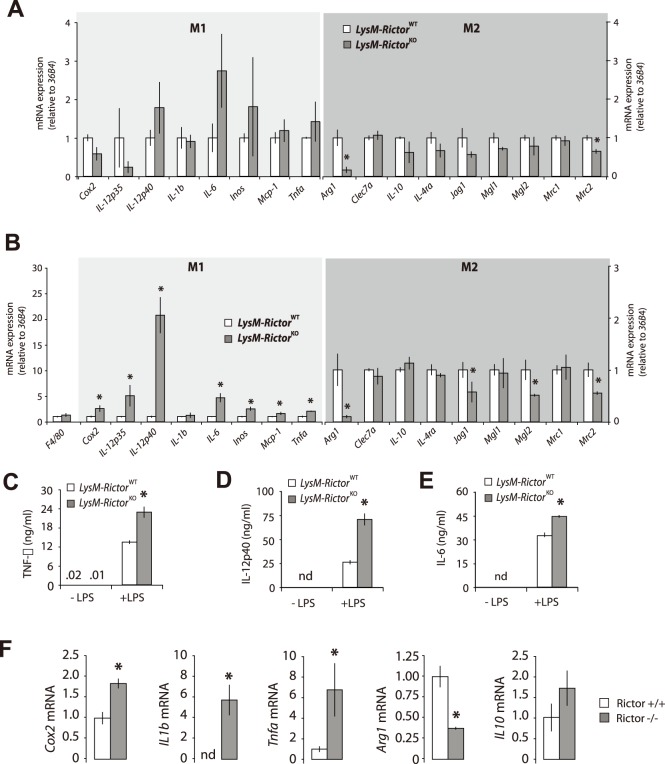
mTORC2 loss exacerbates the pro-inflammatory profile of BMDM in response to LPS. (A) Basal gene expression in macrophages isolated from LysM-*Rictor* ^WT^ or LysM-*Rictor* ^KO^ mice. BMDM were isolated and differentiated for 6 days as described in the methods sections. For each genotype, BMDM isolated from 3 mice were pooled together to get enough biological material for all the studies. Cells were then allowed to rest for 48 hours. mRNA expression of markers of the classically activated (M1) and alternatively activated (M2) macrophages were measured by qRT-PCR and normalized to 36B4 mRNA levels. Data are expressed as the mean ± SEM for n = 3 per condition. *p<0.05 versus control. (B) Impact of LPS on gene expression in macrophages isolated from LysM-*Rictor* ^WT^ or LysM-*Rictor* ^KO^ mice. Cells isolated and differentiated as in A were treated with LPS (250ng/ml) for 10 hours. RNA expression was measured as described in A. Data are expressed as the mean ± SEM for n = 3 per condition. *p<0.05 versus control. This experiment was performed 3 times with similar outcome. (C to E) Secretion of pro-inflammatory cytokines by BMDM isolated from LysM-*Rictor* ^WT^ or LysM-*Rictor* ^KO^ mice in response to LPS. Cell culture media in which BMDM were incubated was collected and (C) TNF-α, (D) IL-12P40 and (E) IL-6 secretion levels were measured by ELISA. This measurement was repeated in another independent experiment and similar results were observed. (F) Impact of LPS on gene expression in *Rictor* ^+/+^ and *Rictor* ^−/−^ MEFs. MEFs were plated 24 hours before the experimentation. The next day, cells were then treated with LPS (5ug/ml) for 10 hours. mRNA expression of several genes was measured by qRT-PCR and normalized to 36B4 mRNA levels. Data are expressed as the mean ± SEM for n = 4 per condition. *p<0.05 versus control. This experiment was repeated once with similar outcome.

**Figure 5:**
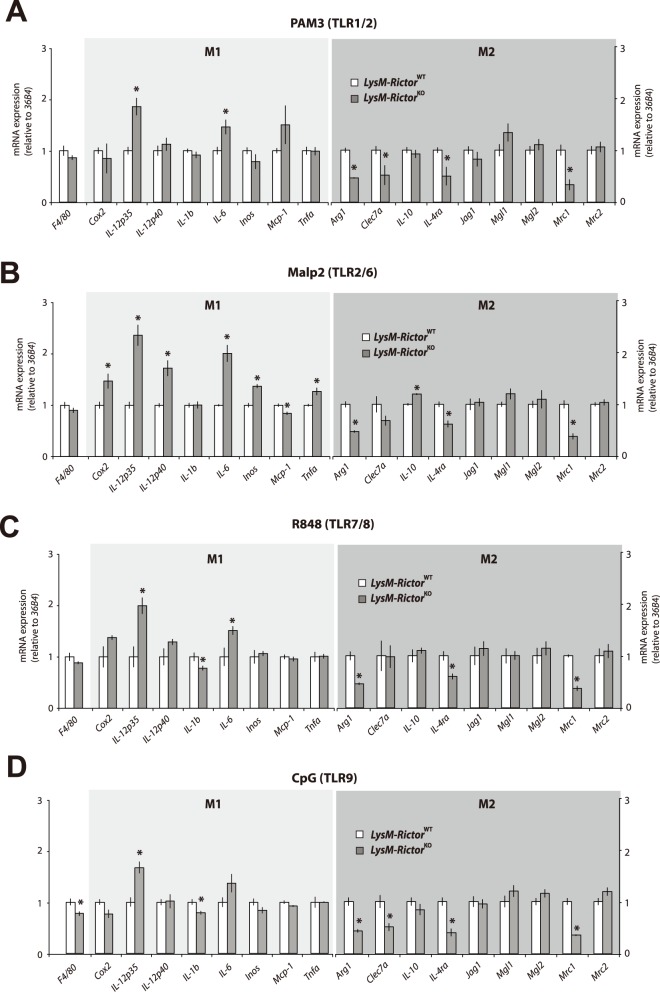
mTORC2 loss exacerbates the pro-inflammatory profile of BMDM in response to several TLR agonists. (A to D) Impact of several TLR agonists on gene expression in macrophages isolated from LysM-*Rictor* ^WT^ or LysM-*Rictor* ^KO^ mice. BMDM were isolated from LysM-*Rictor* ^WT^ or LysM-*Rictor* ^KO^ mice and were differentiated for 6 days *in vitro*. Cells were then plated and incubated 24 hours. Cells were then treated with (A) PAM3 (1ug/ml), (B) MALP2 (0.1ug/ml), (C) R848 (0.1uM), or CpG (0.5uM) for 8 hours. mRNA expression of markers of the classically activated (M1) and alternatively activated (M2) macrophages were measured by qRT-PCR and normalized to 36B4 mRNA levels. Data are expressed as the mean ± SEM for n = 4 per condition. *p<0.05 versus control. This experiment was repeated twice and similar results were observed.

**Figure 6:**
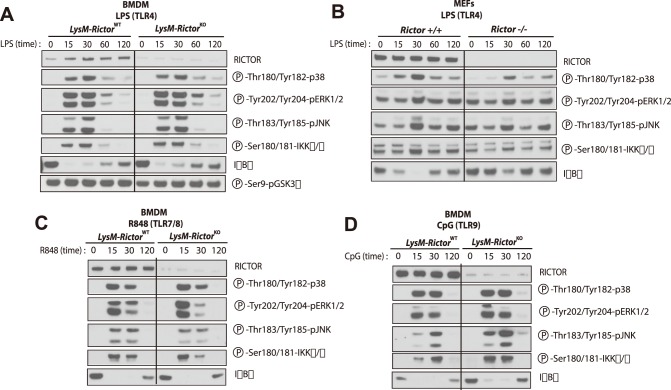
The pro-inflammatory profile induced by *Rictor* loss is not associated with consistent modulations in MAP kinase signalling and IKKα/β activation. (A) Impact of LPS on cell signalling in BMDM isolated from LysM-*Rictor* ^WT^ or LysM-*Rictor* ^KO^ mice. BMDM were isolated from LysM-*Rictor* ^WT^ or LysM-*Rictor* ^KO^ mice and were differentiated for 6 days *in vitro*. Cells were then plated, incubated 24 hours and then treated with LPS (250ng/ml) for the indicated times. Proteins were extracted and lysates were analysed by immunoblotting for indicated proteins. (B) Impact of LPS on cell signalling in *Rictor* ^+/+^ and *Rictor* ^−/−^ MEFs. MEFs were plated 24 hours before the experimentation. The next day, cells were treated with LPS (5ug/ml) for the indicate time. Proteins were extracted and lysates were analysed by immunoblotting for indicated proteins. (C–D) Impact of other TLR agonists on cell signalling in BMDM isolated from LysM-*Rictor* ^WT^ or LysM-*Rictor* ^KO^ mice. Cells were isolated as described in A and were treated with either (C) R848 or (D) CpG for the indicated times. Protein were extracted and treated as described in A.

To determine whether the differential signalling pattern linked to mTORC2 deletion in macrophages translates into changes in cell functions, we measured the expression of numerous genes previously shown to be altered by TLR activation in these cells. First, we investigated the basal gene expression in BMDM cells isolated from either *LysM*-*Rictor* ^WT^ or *LysM*-*Rictor* ^KO^ mice. As shown in Figure 4A, we observed a trend to the heightened expression of proinflammatory mediators expressed by classically activated (M1) macrophages such as *interleukin-12* (*Il-12), interleukin-6 (Il-6), inducible nitric oxide synthase* (*Inos*), and *tumor necrosis factor-α* (*Tnfa*) in *Rictor* null macrophages. Conversely, the expression of anti-inflammatory genes by alternatively activated macrophages (M2) such as *Arginase 1* (*Arg1*) and *mannose receptor 2* (*Mrc2*) was reduced in these cells. In a second setting, stimulation of BMDM with the TLR4 agonist LPS massively exacerbated these differences (Figure 4B). Indeed, the expression of many proinflammatory chemokines and cytokines including *Tnfa*, *Il-12*, *Il-6*, *Inos*, *monocyte chemoattractant protein 1* (*Mcp1*), and *cyclooxygenase 2* (*Cox2*), was significantly increased in BMDM of *LysM*-*Rictor* ^KO^ mice versus *LysM*-*Rictor* ^WT^ mice upon LPS treatment (Figure 4B). We correlated the gene expression data at the protein level through ELISA measurement of TNF-α, IL-12 and IL-6, which showed the same increases in *Rictor* null BMDM (Figure 4C, 4D, and 4E). LPS treatment was also linked to a significant reduction in the expression of M2 markers *Arg1*, *Mrc2*, *protein jagged-1* (*Jag1*) and *macrophage galactose N-acetyl-galactosamine specific lectin 2* (*Mgl2*), which are genes classified as markers of M2-activated macrophages (Figure 4B). Such a gene expression pattern is suggestive of macrophages skewing towards a M1 proinflammatory profile. Similar results were also observed when *Rictor* null MEFs were treated with LPS (Figure 4F). Interestingly, treatment of wild type or *Rictor* null BMDM with TLR1/2 (PAM3), TLR2/6 (MALP2), R848 (TLR7/8) or TLR9 (CpG) ligands induced a similar increase in the expression of M1 markers and a reduction in the expression of M2 markers (Figure 5). These data argue for a conserved role of mTORC2 in the TLR signaling that affect the M1/M2 polarization. Importantly, the modulation of the inflammatory response linked to mTORC2 disruption was not linked to any consistent change in signalling through the MAP kinase, a signalling pathway known to play a key role in promoting the nuclear factor kappa light-chain enhancer of activated B cells (NF-κB) activation and the transcription of several pro-inflammatory genes (Figure 6).

**Figure 7:**
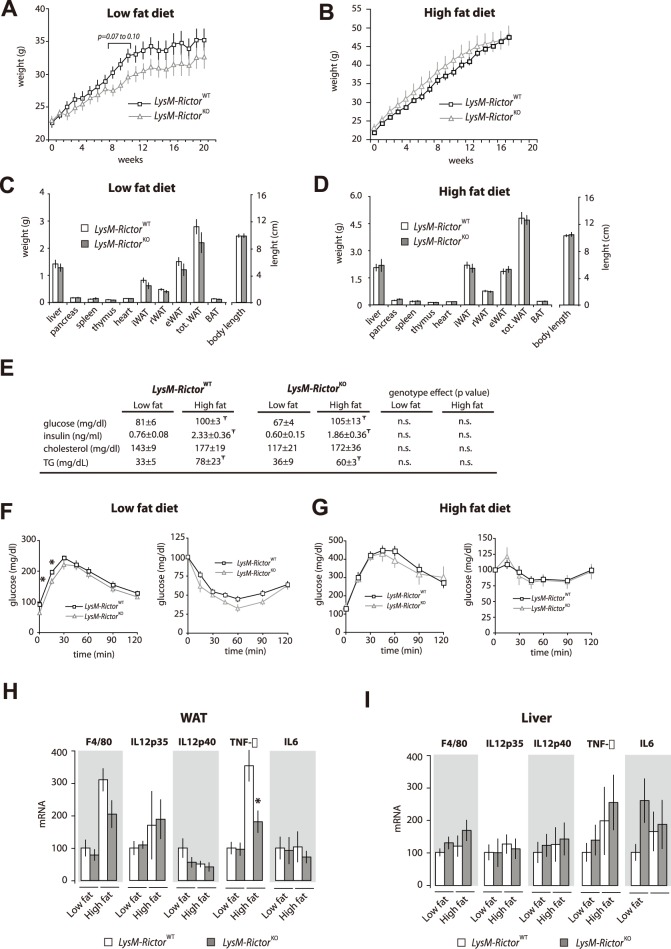
Myeloid-specific deletion of *Rictor* does not affect metabolic homeostasis in mice fed a low or a high-fat diet. (A–D) Body and tissue weight of LysM-*Rictor* ^WT^ or LysM-*Rictor* ^KO^ mice fed a (A and C) low or (B a D) high fat diet for 21 and 23 weeks respectively. (E) Blood parameters of LysM-*Rictor* ^WT^ or LysM-*Rictor* ^KO^ mice fed either a low or a high fat diet. Mice were fasted for 6 hours before blood collection. ^Ŧ^ denotes a significant difference between low fat fed and high fat fed mice (p<0.05). The p value for the genotype effect is indicated on the right side of the table. (F–G) GTT and ITT of LysM-*Rictor* ^WT^ or LysM-*Rictor* ^KO^ mice fed with (F) low or (G) high fat diet. For the panels A to G, n = 6–13 mice per group. (H–I) Gene expression in (H) WAT and (I) liver of LysM-*Rictor* ^WT^ or LysM-*Rictor* ^KO^ mice fed a low or a high fat diet. mRNA expression was measured by qRT-PCR and normalized to 36B4 mRNA levels. Data are expressed as the mean ± SEM for n = 6–10 per condition. ^Ŧ^ denotes a significant difference between low fat fed and high fat fed mice (p<0.05). * denotes a significant difference between LysM-*Rictor* ^WT^ or LysM-*Rictor* ^KO^ mice (p<0.05).

Our findings indicate that mTORC2 is activated upon TLR stimulation and that the loss of complex activity is associated with an exacerbated pro-inflammatory response by macrophages *in vitro*. To test whether myeloid-cell *Rictor* deletion is associated with changes in macrophage function *in vivo*, *LysM*-*Rictor* ^WT^ or *LysM*-*Rictor* ^KO^ mice were either rendered obese, a situation characterized by a chronic low-grade inflammation, or injected with LPS, which mimics an acute, severe septic inflammatory condition. Reports published over the last decade have shown that obesity promotes systemic and low-grade inflammation in humans and rodents and that the inflammatory mediators secreted by activated macrophages play a crucial role in the development of insulin resistance[5], [34]–[38]. Elegant studies have shown that M2 macrophages resides into white adipose tissue (WAT) and that conditions leading to obesity promotes the accumulation of M1 macrophages into WAT[34]. Because our *in vitro* results showed that *Rictor* loss promotes the expression of pro-inflammatory markers while reducing those of anti-inflammatory cytokines, we hypothesized that *LysM*-*Rictor* ^KO^ mice could be prone to inflammation and may become more insulin resistant when exposed to a high fat diet. In order to test this hypothesis, *LysM*-*Rictor* ^WT^ or *LysM*-*Rictor* ^KO^ mice were fed a low or a high fat diet between 21 to 23 weeks. As shown in Figure 7A and 7B, depleting *Rictor* in myeloid cells did not significantly affect the growth curves in response to chow or high fat diet. Consistent with these results, we observed no difference in tissue weight between *LysM*-*Rictor* ^WT^ or *LysM*-*Rictor* ^KO^ mice (Figure 7C and 7D). As expected, feeding mice with a high fat diet increased circulating levels of glucose, insulin, and triglycerides (Figure 7E). However, although *LysM*-*Rictor* ^KO^ fed a chow diet tended to have a lower glycaemia, we observed no significant difference in blood parameters between *LysM*-*Rictor* ^WT^ and *LysM*-*Rictor* ^KO^ mice (Figure 7C and 7D). Supporting these results, glucose and insulin tolerance tests (GTT and ITT respectively) did not reveal any major differences between the genotypes, thus indicating that the depletion of mTORC2 function alone does not deeply affect insulin sensitivity in the context of leanness and obesity *in vivo* (Figure 7F and 7G). Because obesity is known to promote macrophages recruitment into WAT, we measured the expression of inflammatory markers in whole WAT and in the stromal vascular fraction (SVF), which is the cellular compartment into which macrophages reside. As expected from previous reports, we noted a significant increase in the content of the macrophage marker *F4/80* in the WAT of obese mice, such an increase that was similar in *LysM*-*Rictor* ^KO^ and *LysM*-*Rictor* ^WT^ mice (Figure 7H). This was also confirmed histologically (not shown). However, none of the pro-inflammatory genes was increased in *LysM*-*Rictor* ^KO^ mice (Figure 7H). Similar results were obtained when the expression of these genes was tested in the liver (Figure 7I). These results indicate that *Rictor* loss in myeloid cells does not exacerbate the chronic low-grade inflammation and insulin resistance associated to obesity.

**Figure 8:**
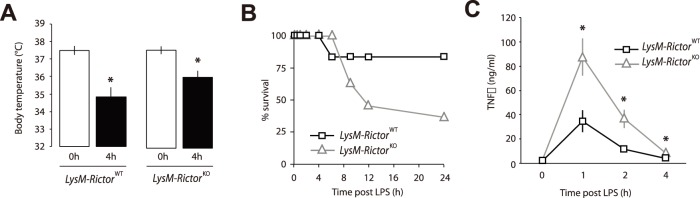
LysM-*Rictor* ^KO^ mice exhibit a higher susceptibility to LPS-induced septic shock. LysM-*Rictor* ^WT^ and LysM-*Rictor* ^KO^mice were injected intraperitoneally with LPS (2.5 mg/kg of body weight) and body temperature was measured 4 hours post-injection. * denotes a significant difference between LysM-*Rictor* ^WT^ or LysM-*Rictor* ^KO^ mice (p<0.05). Data are expressed as the mean ± SEM for n = 6–10 per condition. (B) Survival rate of LysM-*Rictor* ^WT^ or LysM-*Rictor* ^KO^ mice following the injection of LPS. Mice having a core body temperature dropping down to 33.0+/−0.2°C or below following LPS injection were killed. (C) The sensitivity of LysM-*Rictor* ^KO^ mice to LPS-induced hypothermia is linked to an elevation in circulating levels of TNF-α. Plasma was collected from the mice used in the experimentation described in A before LPS injection and 1,2, and 4 hours post injection. TNF-α levels were measured by ELISA. Data are expressed as the mean ± SEM. The graph presented in B is based on the plasma measurements of TNF-α of all LysM-*Rictor* ^WT^ mice (n = 6) vs. the LysM-*Rictor* ^KO^ mice that died over the experiment (n = 7). *p<0.05 versus control.

In a second experimental setting, we assessed the role of mTORC2 in an acute inflammatory context namely an ip injection of the TLR4 agonist LPS. LPS injection in mice leads to endotoxic shock and death. To test whether the loss of mTORC2 could exacerbate inflammation in response to a stimulus mimicking an acute bacterial infection, *LysM*-*Rictor* ^WT^ and *LysM*-*Rictor* ^KO^ mice were ip injected with 2.5 mg/kg of LPS. The body temperature of mice was significantly reduced over the first 4 hours post LPS injection (Figure 8A). Such a hypothermic response has been observed several times in rodents injected with LPS[39]. Preliminary experiments carried in our laboratory showed that mice having a drop in the core body temperature down to 33+/−0.2°C or below following LPS injection have a poor recovery rate and a high probability of death. In order to limit pain, any mouse reaching this limit was humanely sacrificed. Interestingly, we observed that the survival rate of *LysM*-*Rictor* ^KO^ mice was reduced compared to control animals (Figure 8B). Indeed, 64% of *LysM*-*Rictor* ^KO^ mice were sacrificed within 24 hours following LPS injection, compared to only 17% of *LysM*-*Rictor* ^WT^ mice. Importantly, we noted a significant increase in the circulating levels of TNFα in the plasma of *LysM*-*Rictor* ^KO^ mice that did not survive LPS versus the control mice (Figure 8C). The overproduction of TNFα is known as an important factor contributing to hypothermia in response to LPS injection in rodents[40]. These results indicate that, similarly to what observed *in vitro*, the depletion of mTORC2 in macrophages exacerbates inflammation in response to TLR activation *in vivo*.

## Discussion

Although the importance of TLR signalling in the host response to infections has been well demonstrated, an exaggerated inflammatory response can seriously impair the functions of tissues and lead to sceptic shock and death. Additionally, unrestrained activation of TLR can contribute to the development of autoimmune diseases and atherosclerosis and promote the susceptibility to tumor metastasis[4]. These observations indicate that the benefits linked to TLR signalling activation follow a U-shaped curve, where too little activity reduces the ability of the host to fight infection, whereas too much impairs tissue function and systemic homeostasis. The results presented in this report support previous literature showing that the PI3K signalling plays a fundamental role in dampening inflammation in response to TLR activation. Importantly, we show that mTORC2 is required for the maximal activation of Akt in response to TLR ligands and that defects in mTORC2 promotes the skewing of macrophages towards the M1 phenotype and exacerbates the inflammatory response *in vitro* and *in vivo*. This establishes mTORC2 as a key negative regulator of TLR signalling in macrophages and supports the idea that this protein complex could be targeted to modulate excessive inflammatory responses *in vivo*.

Stimulation of myeloid cells with TLR ligands promotes PI3K signalling[13], [17]–[21]. Although several mechanisms linking TLR signalling to PI3K have been proposed[13]–[17], recent reports indicate that B-cell adapter for PI3K (BCAP) plays a key role in this process[13], [17]. BCAP exhibits a constitutive phosphorylation state on tyrosine residues and has the ability to bind the p85 subunit of PI3K [17]. Upon LPS stimulation, a pool of BCAP associated to PI3K is transiently recruited to the plasma membrane, which facilitates PIP3 production by PI3K[17]. From a functional perspective, loss of BCAP severely impairs the activation of PI3K-Akt in response to TLR stimulation and that BCAP deficient mice show an exaggerated pro-inflammatory response to TLR agonists and bacterial infection[13], [17]. Interestingly, these observations are very similar to what we observed here following the deletion of *Rictor*, an essential component of mTORC2. Because mTORC2 activation depends on PI3K, this strongly suggests that mTORC2 in one important mediator of the effect of PI3K on TLR signalling.

mTORC2 is activated by PI3K and, together with PDPK1, regulates the activity of Akt. By directly phosphorylating Akt on Ser473 and Thr308, mTORC2 and PDPK1 insure the maximal activation of this kinase[30], [31]. The functional relevance of Akt in the modulation of TLR stimulation was demonstrated by the findings that direct inhibition of Akt in monocytes promotes a pro-inflammatory phenotype similar to that observed following PI3K inhibition[41]. Supporting these results, *Akt1* deficient macrophages show elevated cytokine production and mice lacking *Akt1* have impaired tolerance to LPS stimulation[23], [24]. It was demonstrated that Akt modulates TLR activation through several mechanisms. Akt phosphorylates and inactivates GSK3β, which reduces NF-KB activation and promotes the expression of the anti-inflammatory cytokine IL-10[41]. Administration of a GSK3β inhibitor, which mimics PI3K-Akt activation, potently suppressed the pro-inflammatory response in mice receiving LPS. Akt has also been shown to reduce inflammation through the transcription factor FoXO1. When active, Akt phosphorylates FoxO1, which promotes its nuclear exclusion[42]. Knockdown or deletion of FoxO1 in various cell type was shown to reduce the expression of pro-inflammatory mediators and to promote the expression of anti-inflammatory cytokines[18], [43]–[46].

Confirming previous findings[13], [17]–[21], we observed that stimulation of myeloid cells with TLR agonists promotes Akt phosphorylation on the sites regulated by both mTORC2 and PDPK1. Importantly, we showed that *Rictor* deletion completely blocks TLR ligand-mediated Akt phosphorylation on Ser473, while Akt phosphorylation on the Thr308 residue was only partially impaired. *Rictor* loss was also associated with a reduction in the activity of SGK1, as illustrated by the reduction in NDRG1 phosphorylation. We observed that the reduction in mTORC2 activity induced by *Rictor* deletion was linked to a reduction in the phosphorylation of FoXO1/3, but not of GSK3β. This observation is particularly interesting considering that FoxO1/3 plays a crucial role in regulating inflammation in response to TLR ligands[43]. Here, we observed that the loss of the essential mTORC2 component *Rictor* in macrophages strongly promoted the expression of pro-inflammatory M1 genes in response to TLR ligands. We also noted a consistent reduction in the expression of several M2 markers in *Rictor* depleted cells. As shown in macrophages lacking BCAP, the pro-inflammatory phenotype linked to *Rictor* deletion was not associated with any consistent modulation of MAP kinase and NF-KB signalling[17]. Interestingly, one recent report revealed that *Rictor* loss in DCs promotes the expression of pro-inflammatory markers in response to TLR4 stimulation, an effect that does not depend on MAP kinases and NF-KB[18]. Brown *et al.* showed that overexpression of a constitutively active Akt blocks the pro-inflammatory phenotype linked to *Rictor* deletion and that the mTORC2-Akt axis regulates inflammation by controlling FoxO1 nuclear localization[18]. Altogether, these observations indicate that mTORC2 is required for the regulation of FoxO1/3 in response to TLR signalling and that this kinase serves as a key negative regulator of inflammation in DCs and macrophages *in vitro*. Our observations also demonstrate a key role of mTORC2 in regulating macrophage polarization.

One important aim of our work was to determine the *in vivo* impact of mTORC2 deletion in macrophages. Our first approach was to induce the development of obesity in control or *LysM*-*Rictor* ^KO^ mice by exposing them to a high fat diet. Obesity is recognized as a low grade but chronic inflammatory disease[38], [47]–[49]. Weight gain promotes macrophage infiltration in WAT, their M1 polarization, and the expression of several pro-inflammatory cytokines[34]–[38], [50]. Free fatty acids, whose concentration is high in obesity, have been shown to activate TLR4 and to promote cytokine production and inflammation[51], [52]. The elevation in cytokine levels causes systemic insulin resistance by reducing the efficiency of the insulin signalling cascade in peripheral tissues[53]. Because *LysM*-*Rictor* ^KO^ macrophages showed an exaggerated response to TLR stimulation *in vitro*, we hypothesized that *LysM*-*Rictor* ^KO^ mice would be prone to inflammation and insulin resistance when chronically fed a high fat diet. Although high fat feeding contributed to promote inflammation, macrophage infiltration in WAT and systemic insulin resistance, we did not observe a significant impact of *Rictor* loss on these parameters. Using a very similar approach, Kawano *et al.* developed a mouse model lacking the other Akt regulating kinase PDPK1 in myeloid cells (*LysM-PDPK1^KO^*)[46]. They observed that exposition of *LysM-PDPK1^KO^* mice to a high fat diet increased macrophages migration to WAT, M1 macrophages polarization, the expression of pro-inflammatory genes in WAT, and insulin resistance[46]. Strikingly, all the effects linked to *Pdpk1* loss were corrected by overexpressing a dominant negative form of FoxO1 in macrophages, indicating that PDPK1 affects inflammation essentially through its effect on the Akt-FoxO1 axis. The results presented by Kawano *et al.* greatly differ from ours. It is possible that the discrepancy between our studies could relate to a different residual Akt activity remaining in myeloid cells following *Rictor* or *Pdpk1* deletion. Here, we observed that the loss of *Rictor* minimally affected the basal phosphorylation of Akt on Thr308, the site regulated by PDPK1. In response to TLR ligands, *LysM*-*Rictor* ^KO^ macrophages showed only a partial defect in Thr308 phosphorylation. Importantly, studies in mTORC2-deficient MEFs have shown that maintenance of Thr308 phosphorylation alone empowers Akt with enough activity to phosphorylate many of its substrates[27], [33], [54]. The fact that PDPK1 loss completely abrogates Thr308 phosphorylation could explain the differences observed between our reports. From a different perspective, we cannot exclude the possibility that differences in the genetic background of mice or variation in the composition of the diet could have altered the severity of the inflammatory responses and the outcome of our studies.

To determine the importance of mTORC2 for the regulation of the inflammatory response in the context of severe and acute infection, we have tested the impact of LPS injection in *LysM*-*Rictor* ^KO^ mice. We observed that deletion of *Rictor* in myeloid cells have exacerbated systemic inflammation following a LPS challenge. The levels of circulating TNFα were significantly higher in mice lacking the mTORC2 component *Rictor*. Moreover, *LysM*-*Rictor* ^KO^ mice showed a more pronounced reduction in body temperature following the LPS challenge. Such hyper responsiveness to LPS was observed in mice lacking PI3K[55], BCAP[13], [17], PDPK1[22], or Akt1[23], [24] and in mice treated with PI3K inhibitors[56]. Conversely, a reduction in inflammation was reported in mice lacking phosphatase and tensin homolog (PTEN), a negative regulator of PI3K signalling[55]. Interestingly, several reports showed that insulin, a very potent activator of PI3K, significantly reduces the severity of endotoxemia in rodents injected with LPS[57], [58]. When combined, these observations clearly show that PI3K signalling plays a crucial role in reducing innate inflammation in response to TLR stimulation *in vivo*. Our findings support this model and further confirm that mTORC2, downstream of PI3K, is involved in this process.

Our *in vivo* experiments with *LysM*-*Rictor* ^KO^ mice revealed that the loss of mTORC2 minimally affected the severity of inflammatory response in obese animals, but had a significant impact in mice exposed to LPS. This indicates that the anti-inflammatory properties of mTORC2 depend on the severity of the inflammatory challenge. These interesting observations support the idea that activation of PI3K signalling by TLR stimulation has evolved to protect the host against the negative impact linked to excessive inflammation. Obesity, which is linked to a low grade but chronic inflammatory state, may not represent a sufficient inflammatory challenge requiring the retro-inhibition of TLR by PI3K signalling. Overall, our results indicate that mTORC2 is a key negative regulator of macrophages TLR signalling and support the idea that this protein complex could be targeted to modulate inflammation, especially in the context of severe inflammatory challenges.

## References

[1] Murray PJ, Wynn TA (2011) Protective and pathogenic functions of macrophage subsets. Nat Rev Immunol 11: 723–737. 21997792 10.1038/nri3073 PMC3422549

[2] Gordon S (2003) Alternative activation of macrophages. Nat Rev Immunol 3: 23–35. 12511873 10.1038/nri978

[3] Kawai T, Akira S (2010) The role of pattern-recognition receptors in innate immunity: update on Toll-like receptors. Nat Immunol 11: 373–384. 20404851 10.1038/ni.1863

[4] O’Neill LA, Bryant CE, Doyle SL (2009) Therapeutic targeting of Toll-like receptors for infectious and inflammatory diseases and cancer. Pharmacol Rev 61: 177–197. 19474110 10.1124/pr.109.001073 PMC2846156

[5] Olefsky JM, Glass CK (2010) Macrophages, inflammation, and insulin resistance. Annu Rev Physiol 72: 219–246. 20148674 10.1146/annurev-physiol-021909-135846

[6] Cantley LC (2002) The phosphoinositide 3-kinase pathway. Science 296: 1655–1657. 12040186 10.1126/science.296.5573.1655

[7] Engelman JA, Cantley LC (2006) The Role of the ErbB Family Members in Non-Small Cell “Lung Cancers Sensitive to Epidermal Growth Factor Receptor Kinase Inhibitors”. Clin Cancer Res 12: 4372s–4376s. 16857813 10.1158/1078-0432.CCR-06-0795

[8] Pearce LR, Komander D, Alessi DR (2010) The nuts and bolts of AGC protein kinases. Nat Rev Mol Cell Biol 11: 9–22. 20027184 10.1038/nrm2822

[9] Manning BD, Cantley LC (2007) AKT/PKB signaling: navigating downstream. Cell 129: 1261–1274. 17604717 10.1016/j.cell.2007.06.009 PMC2756685

[10] Brown J, Wang H, Hajishengallis GN, Martin M (2011) TLR-signaling networks: an integration of adaptor molecules, kinases, and cross-talk. J Dent Res 90: 417–427. 20940366 10.1177/0022034510381264 PMC3075579

[11] Hazeki K, Nigorikawa K, Hazeki O (2007) Role of phosphoinositide 3-kinase in innate immunity. Biol Pharm Bull 30: 1617–1623. 17827709 10.1248/bpb.30.1617

[12] Troutman TD, Bazan JF, Pasare C (2012) Toll-like receptors, signaling adapters and regulation of the pro-inflammatory response by PI3K. Cell Cycle 11: 3559–3567. 22895011 10.4161/cc.21572 PMC3478307

[13] Troutman TD, Hu W, Fulenchek S, Yamazaki T, Kurosaki T, et al (2012) Role for B-cell adapter for PI3K (BCAP) as a signaling adapter linking Toll-like receptors (TLRs) to serine/threonine kinases PI3K/Akt. Proc Natl Acad Sci U S A 109: 273–278. 22187460 10.1073/pnas.1118579109 PMC3252926

[14] Arbibe L, Mira JP, Teusch N, Kline L, Guha M, et al (2000) Toll-like receptor 2-mediated NF-kappa B activation requires a Rac1-dependent pathway. Nat Immunol 1: 533–540. 11101877 10.1038/82797

[15] Sarkar SN, Peters KL, Elco CP, Sakamoto S, Pal S, et al (2004) Novel roles of TLR3 tyrosine phosphorylation and PI3 kinase in double-stranded RNA signaling. Nat Struct Mol Biol 11: 1060–1067. 15502848 10.1038/nsmb847

[16] Rhee SH, Kim H, Moyer MP, Pothoulakis C (2006) Role of MyD88 in phosphatidylinositol 3-kinase activation by flagellin/toll-like receptor 5 engagement in colonic epithelial cells. J Biol Chem 281: 18560–18568. 16644730 10.1074/jbc.M513861200

[17] Ni M, MacFarlane AW, Toft M, Lowell CA, Campbell KS, et al (2012) B-cell adaptor for PI3K (BCAP) negatively regulates Toll-like receptor signaling through activation of PI3K. Proc Natl Acad Sci U S A 109: 267–272. 22187458 10.1073/pnas.1111957108 PMC3252908

[18] Brown J, Wang H, Suttles J, Graves DT, Martin M (2011) Mammalian target of rapamycin complex 2 (mTORC2) negatively regulates Toll-like receptor 4-mediated inflammatory response via FoxO1. J Biol Chem 286: 44295–44305. 22045807 10.1074/jbc.M111.258053 PMC3247956

[19] Guha M, Mackman N (2002) The phosphatidylinositol 3-kinase-Akt pathway limits lipopolysaccharide activation of signaling pathways and expression of inflammatory mediators in human monocytic cells. J Biol Chem 277: 32124–32132. 12052830 10.1074/jbc.M203298200

[20] McGuire VA, Gray A, Monk CE, Santos SG, Lee K, et al (2013) Cross Talk between the Akt and p38alpha Pathways in Macrophages Downstream of Toll-Like Receptor Signaling. Mol Cell Biol 33: 4152–4165. 23979601 10.1128/MCB.01691-12 PMC3811899

[21] Ojaniemi M, Glumoff V, Harju K, Liljeroos M, Vuori K, et al (2003) Phosphatidylinositol 3-kinase is involved in Toll-like receptor 4-mediated cytokine expression in mouse macrophages. Eur J Immunol 33: 597–605. 12616480 10.1002/eji.200323376

[22] Chaurasia B, Mauer J, Koch L, Goldau J, Kock AS, et al (2010) Phosphoinositide-dependent kinase 1 provides negative feedback inhibition to Toll-like receptor-mediated NF-kappaB activation in macrophages. Mol Cell Biol 30: 4354–4366. 20584979 10.1128/MCB.00069-10 PMC2937543

[23] Arranz A, Doxaki C, Vergadi E, Martinez de la Torre Y, Vaporidi K, et al (2012) Akt1 and Akt2 protein kinases differentially contribute to macrophage polarization. Proc Natl Acad Sci U S A 109: 9517–9522. 22647600 10.1073/pnas.1119038109 PMC3386059

[24] Androulidaki A, Iliopoulos D, Arranz A, Doxaki C, Schworer S, et al (2009) The kinase Akt1 controls macrophage response to lipopolysaccharide by regulating microRNAs. Immunity 31: 220–231. 19699171 10.1016/j.immuni.2009.06.024 PMC2865583

[25] Laplante M, Sabatini DM (2012) mTOR signaling in growth control and disease. Cell 149: 274–293. 22500797 10.1016/j.cell.2012.03.017 PMC3331679

[26] Zoncu R, Efeyan A, Sabatini DM (2011) mTOR: from growth signal integration to cancer, diabetes and ageing. Nat Rev Mol Cell Biol 12: 21–35. 21157483 10.1038/nrm3025 PMC3390257

[27] Guertin DA, Stevens DM, Thoreen CC, Burds AA, Kalaany NY, et al (2006) Ablation in mice of the mTORC components raptor, rictor, or mLST8 reveals that mTORC2 is required for signaling to Akt-FOXO and PKCalpha, but not S6K1. Dev Cell 11: 859–871. 17141160 10.1016/j.devcel.2006.10.007

[28] Shiota C, Woo JT, Lindner J, Shelton KD, Magnuson MA (2006) Multiallelic disruption of the rictor gene in mice reveals that mTOR complex 2 is essential for fetal growth and viability. Dev Cell 11: 583–589. 16962829 10.1016/j.devcel.2006.08.013

[29] Clausen BE, Burkhardt C, Reith W, Renkawitz R, Forster I (1999) Conditional gene targeting in macrophages and granulocytes using LysMcre mice. Transgenic Res 8: 265–277. 10621974 10.1023/a:1008942828960

[30] Alessi DR, James SR, Downes CP, Holmes AB, Gaffney PR, et al (1997) Characterization of a 3-phosphoinositide-dependent protein kinase which phosphorylates and activates protein kinase Balpha. Curr Biol 7: 261–269. 9094314 10.1016/s0960-9822(06)00122-9

[31] Sarbassov DD, Guertin DA, Ali SM, Sabatini DM (2005) Phosphorylation and regulation of Akt/PKB by the rictor-mTOR complex. Science 307: 1098–1101. 15718470 10.1126/science.1106148

[32] Garcia-Martinez JM, Alessi DR (2008) mTOR complex 2 (mTORC2) controls hydrophobic motif phosphorylation and activation of serum- and glucocorticoid-induced protein kinase 1 (SGK1). Biochem J 416: 375–385. 18925875 10.1042/BJ20081668

[33] Jacinto E, Facchinetti V, Liu D, Soto N, Wei S, et al (2006) SIN1/MIP1 maintains rictor-mTOR complex integrity and regulates Akt phosphorylation and substrate specificity. Cell 127: 125–137. 16962653 10.1016/j.cell.2006.08.033

[34] Lumeng CN, Bodzin JL, Saltiel AR (2007) Obesity induces a phenotypic switch in adipose tissue macrophage polarization. J Clin Invest 117: 175–184. 17200717 10.1172/JCI29881 PMC1716210

[35] Nomiyama T, Perez-Tilve D, Ogawa D, Gizard F, Zhao Y, et al (2007) Osteopontin mediates obesity-induced adipose tissue macrophage infiltration and insulin resistance in mice. J Clin Invest 117: 2877–2888. 17823662 10.1172/JCI31986 PMC1964510

[36] Cancello R, Tordjman J, Poitou C, Guilhem G, Bouillot JL, et al (2006) Increased infiltration of macrophages in omental adipose tissue is associated with marked hepatic lesions in morbid human obesity. Diabetes 55: 1554–1561. 16731817 10.2337/db06-0133

[37] Xu H, Barnes GT, Yang Q, Tan G, Yang D, et al (2003) Chronic inflammation in fat plays a crucial role in the development of obesity-related insulin resistance. J Clin Invest 112: 1821–1830. 14679177 10.1172/JCI19451 PMC296998

[38] Wellen KE, Hotamisligil GS (2003) Obesity-induced inflammatory changes in adipose tissue. J Clin Invest 112: 1785–1788. 14679172 10.1172/JCI20514 PMC297006

[39] Habicht GS (1981) Body temperature in normal and endotoxin-treated mice of different ages. Mech Ageing Dev 16: 97–104. 7253723 10.1016/0047-6374(81)90037-3

[40] Bauss F, Droge W, Mannel DN (1987) Tumor necrosis factor mediates endotoxic effects in mice. Infect Immun 55: 1622–1625. 3596805 10.1128/iai.55.7.1622-1625.1987 PMC260568

[41] Martin M, Rehani K, Jope RS, Michalek SM (2005) Toll-like receptor-mediated cytokine production is differentially regulated by glycogen synthase kinase 3. Nat Immunol 6: 777–784. 16007092 10.1038/ni1221 PMC1933525

[42] Calnan DR, Brunet A (2008) The FoxO code. Oncogene 27: 2276–2288. 18391970 10.1038/onc.2008.21

[43] Fan W, Morinaga H, Kim JJ, Bae E, Spann NJ, et al (2010) FoxO1 regulates Tlr4 inflammatory pathway signalling in macrophages. EMBO J 29: 4223–4236. 21045807 10.1038/emboj.2010.268 PMC3018786

[44] Siqueira MF, Li J, Chehab L, Desta T, Chino T, et al (2010) Impaired wound healing in mouse models of diabetes is mediated by TNF-alpha dysregulation and associated with enhanced activation of forkhead box O1 (FOXO1). Diabetologia 53: 378–388. 19902175 10.1007/s00125-009-1529-y PMC3130195

[45] Su D, Coudriet GM, Hyun Kim D, Lu Y, Perdomo G, et al (2009) FoxO1 links insulin resistance to proinflammatory cytokine IL-1beta production in macrophages. Diabetes 58: 2624–2633. 19651810 10.2337/db09-0232 PMC2768186

[46] Kawano Y, Nakae J, Watanabe N, Fujisaka S, Iskandar K, et al (2012) Loss of Pdk1-Foxo1 signaling in myeloid cells predisposes to adipose tissue inflammation and insulin resistance. Diabetes 61: 1935–1948. 22586579 10.2337/db11-0770 PMC3402298

[47] Cancello R, Clement K (2006) Is obesity an inflammatory illness? Role of low-grade inflammation and macrophage infiltration in human white adipose tissue. Bjog 113: 1141–1147. 16903845 10.1111/j.1471-0528.2006.01004.x

[48] Cusi K (2010) The role of adipose tissue and lipotoxicity in the pathogenesis of type 2 diabetes. Curr Diab Rep 10: 306–315. 20556549 10.1007/s11892-010-0122-6

[49] Ouchi N, Parker JL, Lugus JJ, Walsh K (2011) Adipokines in inflammation and metabolic disease. Nat Rev Immunol 11: 85–97. 21252989 10.1038/nri2921 PMC3518031

[50] Weisberg SP, McCann D, Desai M, Rosenbaum M, Leibel RL, et al (2003) Obesity is associated with macrophage accumulation in adipose tissue. J Clin Invest 112: 1796–1808. 14679176 10.1172/JCI19246 PMC296995

[51] Pal D, Dasgupta S, Kundu R, Maitra S, Das G, et al (2012) Fetuin-A acts as an endogenous ligand of TLR4 to promote lipid-induced insulin resistance. Nat Med 18: 1279–1285. 22842477 10.1038/nm.2851

[52] Shi H, Kokoeva MV, Inouye K, Tzameli I, Yin H, et al (2006) TLR4 links innate immunity and fatty acid-induced insulin resistance. J Clin Invest 116: 3015–3025. 17053832 10.1172/JCI28898 PMC1616196

[53] Hotamisligil GS (2006) Inflammation and metabolic disorders. Nature 444: 860–867. 17167474 10.1038/nature05485

[54] Hietakangas V, Cohen SM (2007) Re-evaluating AKT regulation: role of TOR complex 2 in tissue growth. Genes Dev 21: 632–637. 17369395 10.1101/gad.416307 PMC1820936

[55] Luyendyk JP, Schabbauer GA, Tencati M, Holscher T, Pawlinski R, et al (2008) Genetic analysis of the role of the PI3K-Akt pathway in lipopolysaccharide-induced cytokine and tissue factor gene expression in monocytes/macrophages. J Immunol 180: 4218–4226. 18322234 10.4049/jimmunol.180.6.4218 PMC2834303

[56] Schabbauer G, Tencati M, Pedersen B, Pawlinski R, Mackman N (2004) PI3K-Akt pathway suppresses coagulation and inflammation in endotoxemic mice. Arterioscler Thromb Vasc Biol 24: 1963–1969. 15319270 10.1161/01.ATV.0000143096.15099.ce

[57] Zou B, Chen Q, Tang S, Gao T, Zhang J, et al (2012) Timing of insulin therapy affects the inflammatory response in endotoxemic rats. Inflammation 35: 723–729. 21809046 10.1007/s10753-011-9367-8

[58] Jeschke MG, Klein D, Bolder U, Einspanier R (2004) Insulin attenuates the systemic inflammatory response in endotoxemic rats. Endocrinology 145: 4084–4093. 15192048 10.1210/en.2004-0592

